# Privacy-Preserving Detection of Post-Fall Lying Posture Using a Low-Resolution Infrared Sensor and an Edge FOMO Neural Network

**DOI:** 10.3390/s26185868

**Published:** 2026-09-16

**Authors:** Michaela Mrazkova, Jakub Vanek, Martin Faltus, Vit Janovsky

**Affiliations:** University Centre for Energy Efficient Buildings, Czech Technical University in Prague, 273 43 Bustehrad, Czech Republic; jakub.vanek@cvut.cz (J.V.); martin.faltus@cvut.cz (M.F.); vit.janovsky@cvut.cz (V.J.)

**Keywords:** post-fall detection, long-lie detection, lying-posture recognition, thermal imaging, low-resolution infrared, FOMO, edge AI, TinyML, OpenMV, Lepton 3.1R, ambient assisted living, privacy-preserving

## Abstract

Falls in older adults are a leading cause of injury, and the time spent on the floor afterwards is the stronger predictor of outcome. We present a low-cost system that detects the sustained lying posture following a fall. An FLIR Lepton 3.1R (160 × 120 px) and an int8-quantized FOMO detector run fully on an OpenMV RT1062 board; no image data leaves the node. Ten healthy adults, none in the training data, followed a structured posture protocol, yielding 3034 labelled frames. No fall, real or simulated, was recorded: lying is a proxy for a post-fall state, and claims are restricted accordingly. The detector produced an output on only 66.3% of labelled frames, with a strong class dependence (75.6% lying, 51.7% standing). End-to-end, binary lying-posture recognition reached a sensitivity of 0.723 (95% CI 0.637–0.812) and an F1 of 0.801. Every sustained lying bout of at least 20 s was flagged, but the deployed alert rule also produced roughly one hundred false alerts per hour of non-lying activity. Low-resolution thermal sensing is therefore a workable basis for long-lie detection; the limiting factors are the detector’s class-dependent miss rate and the alert logic, not the posture classifier.

## 1. Introduction

Falls are one of the leading causes of injury and death among older adults, making their reliable and timely detection a key requirement in health monitoring. Delayed assistance following a fall can significantly increase the risk of serious complications, particularly in cases of prolonged immobility [1,2,3]. Current approaches to fall detection are typically divided into wearable and non-contact devices. Wearable solutions based on inertial sensors such as accelerometers and gyroscopes can achieve high detection accuracy under controlled conditions, often using machine learning or deep learning techniques [4,5,6]. However, their performance in real-world scenarios is limited by user compliance and the similarity between falls and normal daily activities, which leads to false detections. These limitations drive the development of contactless monitoring systems that do not rely on user interaction.

Fall detection systems based on visual sensing, particularly those that use RGB cameras in combination with deep learning architectures such as convolutional neural networks, have demonstrated high effectiveness in recognizing human posture and movement [7]. Despite their effectiveness, however, these approaches raise significant privacy concerns, as they capture identifiable visual information and are often unsuitable for continuous monitoring in sensitive environments such as homes or healthcare facilities. Furthermore, their performance can be affected by challenging lighting conditions, obstacles, and changes in the environment. Alternative sensing methods, including radar-based systems, address privacy concerns by avoiding the capture of visual data. However, these systems are typically associated with higher costs, greater system complexity, and more demanding signal processing requirements [8]. As a result, there is a growing need for sensor-based approaches that can balance detection performance, privacy protection, and practical usability in real-world applications.

Infrared (IR) and thermal imaging technologies offer a promising compromise because they detect only thermal signatures, thereby naturally preserving user anonymity while enabling operation in low-light or dark environments [9,10]. Previous studies have demonstrated the utility of thermal imaging for person detection and activity recognition, including fall-detection scenarios [11,12]. However, existing infrared-based approaches are often limited by low spatial resolution, sensitivity to ambient-temperature fluctuations and insufficient focus on real-time deployment in resource-constrained environments. Furthermore, many existing systems prioritize detection accuracy without addressing practical constraints such as computational efficiency, system cost and ease of integration into real-world environments.

The goal of this study is to design and experimentally validate a low-cost, privacy-preserving system for detecting the sustained lying posture that follows a fall, using low-resolution infrared imaging and on-device inference. We deliberately frame the task as post-fall lying-posture detection rather than fall detection. The clinically consequential quantity after a fall is the time spent on the floor before help arrives: among people over 90, the inability to get up is associated with markedly worse outcomes independently of the injury sustained in the fall itself [1]. A long-lie decision window of minutes is also far more tolerant of transient misclassification during posture transitions than an impact-detection window of milliseconds, which matters for a sensor operating at approximately 2 Hz. The proposed system combines an FLIR Lepton 3.1R thermal sensor with an embedded OpenMV RT1062 platform and a lightweight FOMO (Faster Objects, More Objects) detector, enabling real-time inference directly on the device without cloud processing [13]. FOMO is a fully convolutional detector derived from the MobileNetV2 backbone [14] that predicts class centroids on a coarse feature map instead of bounding boxes; this removes the anchor and box-regression machinery and makes it one of the few object-detection architectures that fits in the memory budget of a Cortex-M-class microcontroller [15]. Of the three limitations of prior infrared work identified above, this study addresses one—the lack of demonstrated real-time, fully on-device deployment—and does so at a spatial resolution of 160 × 120 px (19,200 px), which is one to two orders of magnitude higher than the 8 × 8 to 32 × 24 arrays used in most low-cost infrared fall work but far below that of a conventional camera. Ambient-temperature robustness and ultra-low-resolution operations are explicitly not addressed and are treated as limitations. The system is evaluated using a structured experimental protocol that includes both static poses and activities of daily living, with performance assessed at the frame level, at the event level, and over the operating range of the detector confidence threshold.

## 2. Materials and Methods

### 2.1. Hardware Platform

The sensor platform was built using the OpenMV Cam RT1062 development board (OpenMV, LLC, Atlanta, GA, USA; NXP i.MX RT1062, NXP Semiconductors, Eindhoven, The Netherlands; Cortex-M7 @ 600 MHz, 16 MB SDRAM, 16 MB flash) in combination with an FLIR Lepton 3.1R (Teledyne FLIR, Wilsonville, OR, USA) for capturing long-wave infrared (LWIR) radiation, connected via the official OpenMV Lepton expansion module [16]. The Lepton 3.1R is an uncooled microbolometer with a resolution of 160 × 120 pixels, operating in the 8–14 um spectral range, with a noise-equivalent temperature difference (NETD) below 50 mK, a horizontal field of view of 95 degrees, a radiometric scene range of −10 to +140 degrees C and a frame rate limited by the VoSPI interface to approximately 8.7 Hz [17]. The camera was mounted on a fixed bracket in the corner of an 8.0 m × 5.0 m room at a height of approximately 2.65 m, with the optical axis inclined approximately 30 degrees below the horizontal, giving an oblique downward view of the floor. Three consequences of this geometry are used in Section 3.2 and are stated here. The 320 × 240 to 240 × 240 crop reduces the effective horizontal field of view from 95 to approximately 79 degrees, so a single corner-mounted node does not cover the full 90-degree corner. The bottom edge of the frame corresponds to a floor point approximately 1.0 m from the node, so a subject closer than that is below the frame entirely. And over all detected frames, the subject-to-sensor distance, reconstructed from the centroid row under a pinhole model, had a median of 2.8 m and a fifth to 95th percentile range of 1.7–7.5 m.

The complete sensor node is powered over USB-C; its bill of materials and total cost are given in Table 1. All inference runs on the device. No image, video or thermal frame is written to persistent storage or transmitted at any point, and the node was operated throughout with no network connection and no radio attached. The alert output as built is a state change in the on-board indicator LEDs together with a single line on the USB serial console; that console stream is also the source of the per-frame logs analyzed in Section 3.

### 2.2. Data Acquisition and Annotation

Thermal data were collected directly using an OpenMV Cam RT1062 camera via a custom script written in MicroPython, with the imaging pipeline described in Section 2.1. For model training, the frames were converted to normalized grayscale thermal images, while absolute temperature values were retained solely for validation and quality control. The training set was collected incrementally from two subjects, neither of whom appears in the evaluation cohort, and under varying viewing angles, distances from the sensor and body orientations. It comprises 3127 images: 1291 standing, 1512 lying and 157 sitting. Training and validation subsets were formed by a random 80/20 split applied within each class, preserving the class proportions in both subsets.

All images were manually annotated using a custom verification tool and subsequently imported into the Edge Impulse platform. Three classes were defined: standing, sitting and lying. Frames showing ambiguous or transitional positions, frames in which the subject was partially outside the field of view, and frames acquired during initial sensor stabilization were excluded from the labelled set. The same procedure and exclusion rules were applied to the evaluation recordings of Section 2.4, where they remove 252 calibration frames and 673 transitional frames from the 3959 recorded. Annotation was performed by a single annotator; no subset was independently re-annotated, so no inter-rater agreement statistic is available.

### 2.3. Model and Training

The model for postural classification was developed using the Edge Impulse platform (Edge Impulse Inc., San Jose, CA, USA) and is based on the FOMO (Faster Objects, More Objects) architecture [18]. FOMO is a lightweight, fully convolutional neural network derived from MobileNetV2, designed for devices with limited resources. Instead of predicting bounding boxes, the model generates class centroids on a low-resolution feature map, which significantly reduces computational and memory requirements and makes it suitable for deployment in microcontroller-based systems. The network’s input consisted of single-channel thermal images resized to 96 × 96 pixels and normalized separately for each image. The model was trained to classify three body position classes: standing, sitting, and lying. Training was performed over 50 epochs with a batch size of 128 and a learning rate of 0.015. These are the Edge Impulse defaults for this architecture and input size; they were not tuned, and no hyperparameter search was performed. During training, data augmentation techniques, including random transformations and intensity variations, were used to improve the model’s robustness against variations in pose, orientation, and thermal contrast.

Training was performed on the GPU backend provided by the Edge Impulse platform. The final model was quantized to an eight-bit integer format (int8) to enable efficient inference directly on the device. This quantization reduces memory and computational requirements while maintaining sufficient classification accuracy for the given application. The trained model was deployed on an OpenMV Cam RT1062, where it performs real-time inference directly on incoming thermal images. For each image, the model generates class probabilities, and the class with the highest confidence is selected as the predicted class. This lightweight design enables continuous operation on embedded hardware without the need for external processing or data transfer.

The final int8 model occupies 56 kB of flash, of which 20 kB are weights, and requires approximately 230 kB of RAM for intermediate activations at inference time. Footprint, latency and energy are the quantities against which TinyML systems are compared [19]. Its input is a single-channel 96 × 96 image and its output a 12 × 12 grid of four channels, one for background and one for each posture class. Inference, FOMO post-processing and the alert state update of Section 2.5 all execute on the microcontroller, with no host and no network connection.

### 2.4. Experimental Protocol

The performance of the proposed system was evaluated on an independent dataset obtained from ten healthy adult volunteers (ID1-ID10). None of these participants contributed to the training dataset. All participants provided informed consent prior to participation. The cohort comprised ten healthy adults, who were all staff of the host research centre, aged approximately 30 to 50 years, between approximately 165 and 180 cm in height and of average to athletic build; two were women and eight men. Anthropometric data were not recorded at the time of the recordings, so these figures are approximate. All participants wore ordinary indoor clothing. Recordings were made in a single indoor room at an ambient air temperature of approximately 24 degrees C.

Each participant followed a structured protocol of eight steps: (1) initial calibration period with the subject outside the field of view; (2) quiet standing (approximately 20 s); (3) lying down on the floor; (4) returning to a standing position; (5) rapid alternating stand-to-lie transitions; (6) activities of daily living, including walking across the monitored area and bending to pick up an object from the floor; (7) sustained supine lying; and (8) sustained lateral lying. In step 3, the descent was performed voluntarily and under full control, at a self-selected and unhurried pace.

Each recording session lasted approximately 3 min and was conducted in the monitored area described in Section 2.1. The protocol was designed to include both sustained postures and rapid posture changes, and to include movements such as bending that are known to challenge posture-based methods. It is essential to state what the protocol does not contain: no fall, real or simulated, occurs anywhere in this dataset. Every horizontal posture in the recordings is the endpoint of a voluntary, controlled descent. Voluntary lying down and a genuine fall differ in velocity, control, intent and terminal posture, and a fall caused by tripping typically involves substantial horizontal displacement that may carry the subject outside the field of view of a single corner-mounted node. The results reported below therefore quantify the recognition of the lying posture that a fall leaves behind and are not comparable with studies that recorded fall events. All recordings were manually reviewed and annotated following the procedure of Section 2.2.

### 2.5. On-Device Alert Logic

The alert that the node emits is produced by a two-state latching machine evaluated at every inference step. It is specified here in full, because all event-level results in Section 3 depend on it. An FOMO centroid is accepted only if its confidence is at least MIN_CONF = 0.70; all lower-confidence blobs are discarded and never reach the alert logic. Accepted centroids of class lying are treated as fall-like, and centroids of class standing or sitting as clearing. Starting from the idle state, the alert is raised once a fall-like centroid has been observed continuously for FALL_CONFIRM = 200 ms; a fall-free interval longer than FALL_GAP_TOLERANCE = 300 ms resets the confirmation timer. If a fall-like and a clearing centroid appear in the same frame, the fall-like class takes priority and the alert is raised immediately, bypassing the confirmation window. Once raised, the alert latches: no further alert can be generated until it has been cleared, which requires a clearing centroid to be observed continuously for CLEAR_CONFIRM = 400 ms, with a gap tolerance of CLEAR_GAP_TOLERANCE = 300 ms. An alert event is defined as a rising edge of this state.

One consequence of these settings deserves emphasis, because it drives the false-alert behaviour reported in Section 3.5. Frames were logged at a median interval of 470 ms, which is longer than FALL_CONFIRM; the rate at which the state machine itself was evaluated on the device was not measured separately, and the two are not assumed to be equal. What can be stated from the recordings is that the device raised alerts on isolated logged lying frames, so the confirmation window as deployed provides very little temporal filtering. The effect of lengthening this window is quantified in Section 3.5.

### 2.6. Evaluation Definitions and Statistical Treatment

Terminology. Two frame subsets are used throughout. Labelled frames are the 3034 frames carrying an unambiguous static-posture ground truth (standing, sitting or lying); frames annotated as transition or ignore are excluded. Detected frames are the 2010 labelled frames in which the FOMO detector produced at least one centroid above MIN_CONF. Metrics computed over labelled frames are termed end-to-end, since a non-detection is scored as an error; metrics computed over detected frames are termed conditional. Every metric in Section 3 states which of the two denominators it uses.

Non-detection convention. In the binary lying-versus-not-lying analysis, a labelled frame with no detection is scored as a not-lying prediction, matching the deployed alert logic, which raises no alert when nothing is detected. Conditional metrics are reported alongside throughout, so that detector failures and classifier errors can be separated.

Event definitions. A lying episode is a maximal run of consecutive frames annotated as lying, with runs separated by less than 2 s merged into a single episode. An episode counts as an event true positive if at least one alert rising edge falls in the window from 2 s before its onset to 2 s after its offset, and as an event false negative otherwise; an alert falling outside every such window is an event false positive. Detection latency is the interval between episode onset and the first matching alert. False-alert rates are reported per hour of non-lying recorded time.

Statistical treatment. Pooled estimates are reported with 95% percentile confidence intervals from a subject-level bootstrap, in which the ten subjects are resampled with replacement B = 5000 times and each metric is recomputed on the pooled frames of the resampled cohort. Per-subject values are reported alongside every pooled estimate. Two sensitivity analyses accompany the main results: the detector confidence threshold is swept over its reachable range (Section 3.4), and the two free parameters of the alert state machine are varied by re-running the state machine over the recorded frames (Section 3.5). No hypothesis test is performed and no *p*-value is reported.

Distance conversion. Centroid positions are converted to floor distances using the mounting geometry of Section 2.1 under a pinhole camera model. The conversion is class-specific, since the thermal centroid of a standing adult sits approximately 1.0 m above the floor against approximately 0.15 m for a person lying down. The resulting distances are indicative rather than surveyed.

## 3. Results

### 3.1. Dataset Statistics

Across the ten subjects, 3959 frames were recorded over a total of 31.4 min of active protocol (Table 2). The average per-subject recording duration was 188.4 s and the median interval between logged frames was 470 ms, with an effective logging rate of 2.13 Hz; the on-device inference rate was not measured separately. After excluding the 252 calibration frames (ground-truth ignore) and the 673 frames falling inside an annotated posture transition, 3034 labelled frames remained. Of those, 2010 (66.3%) contained an FOMO detection. Following the terminology of Section 2.6, conditional three-class results are reported on the 2010 detected frames (Section 3.2) and end-to-end binary results on all 3034 labelled frames (Section 3.3).

The class distribution of the labelled frames (Table 3) is dominated by lying (60.7%), which is a direct consequence of a protocol containing three lying segments (steps 3, 7 and 8) against a single 20 s standing segment (step 2), a shorter standing window in step 4 and the activities-of-daily-living segment in step 6. The stand class is represented by 1190 labelled frames and the sit class by a single frame, because sitting was not a scheduled posture; sit is reported in the confusion matrix for completeness only and is excluded from all quantitative metrics. This class prior is favourable relative to real monitoring, where lying is rare, and its consequences for the transferability of the reported precision are discussed in Section 4.

### 3.2. Frame-Level Classification

Detection rate. The detector produced an output on 2010 of the 3034 labelled frames (66.3%, 95% CI 0.608–0.720); the remaining 1024 labelled frames (33.7%) carry no detection at all. This detection rate is a first-class result rather than a pre-processing detail, and it is strongly class-dependent (Table 4). A lying person was detected in 75.6% of lying frames, but a standing person in only 51.7% of standing frames. Non-detections corresponded disproportionately to the standing class, whose compact, vertically oriented thermal signature produces a weaker response on the coarse FOMO output grid than the extended signature of a lying body. Other explanations are conceivable—subjects near the edge of the field of view, partially occluded, or in rapid motion—but none of them can be tested here, because the firmware logs a centroid only when the detector fires, so the position of the subject during a non-detection was never recorded. They remain plausible but unverified, and are not presented as findings.

**Figure 1:**
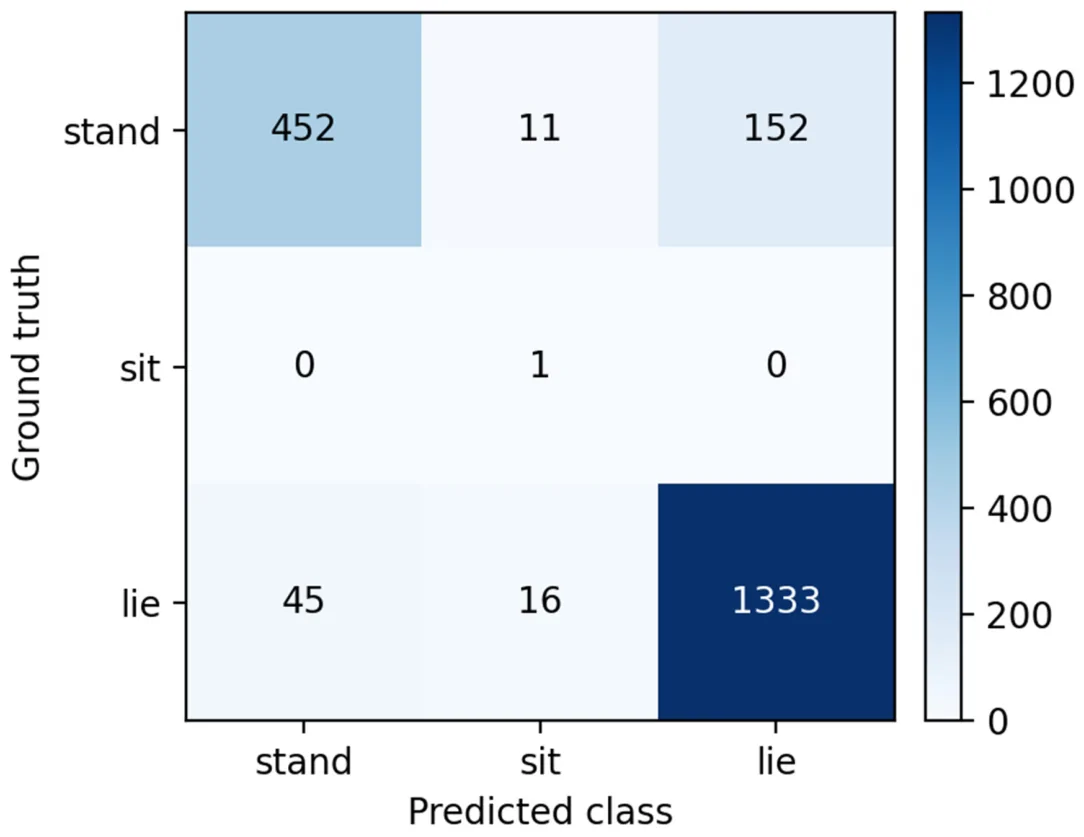
Three-class confusion matrix on the 2010 detected frames across all ten subjects. Columns are FOMO predictions; rows are annotator ground truth. The matrix is conditional on a detection having occurred; the 1024 labelled frames with no detection are not represented here and are accounted for separately in Table 4. The dominant failure mode is stand to lie (152 frames), mostly during the fast stand–lie alternation of protocol step 5 and during the bending action of step 6.

**Figure 2:**
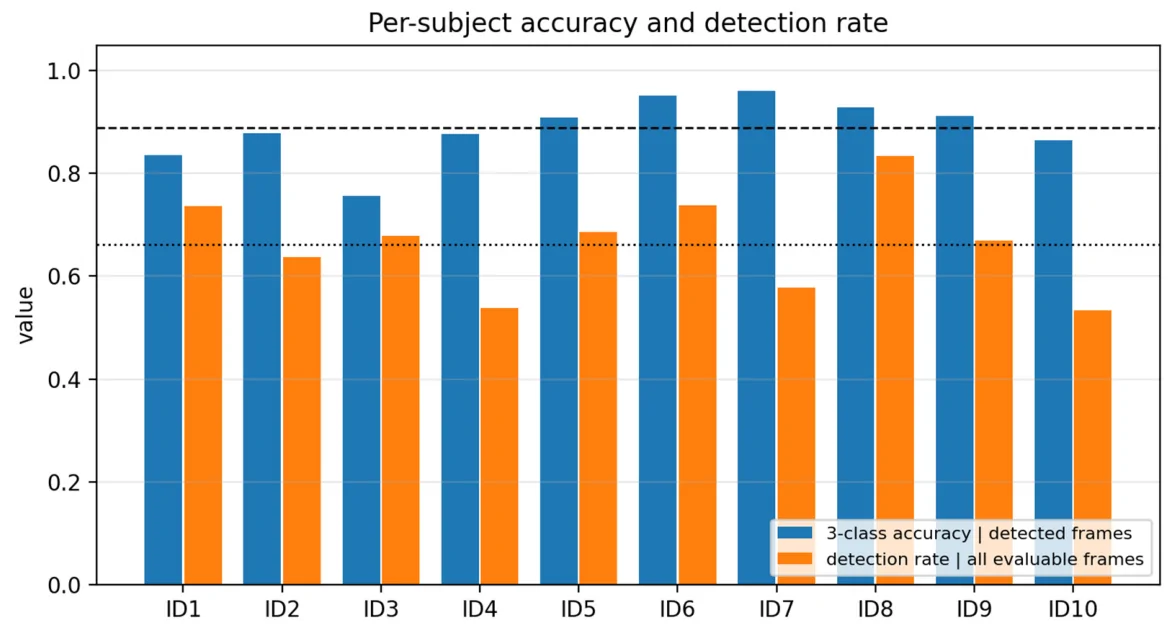
Per-subject conditional three-class accuracy (left bars) and detection rate over all labelled frames (right bars). The dashed line marks the pooled conditional accuracy (0.889) and the dotted line the pooled detection rate (0.662). The two quantities vary largely independently across subjects (accuracy 0.756–0.960, detection rate 0.533–0.834), which is why both are reported: a high accuracy on the frames of a subject’s detector which happened to fire on says nothing about how often it fired.

Conditional three-class performance. On the 2010 detected frames, the model achieved an overall three-class accuracy of 88.9% (95% CI 0.850–0.922). The confusion matrix is shown in Figure 1 and per-class metrics in Table 5; per-subject values are given in Figure 2 and Table 2. The lying class achieved an F1 score of 0.93 (precision 0.90, recall 0.96) and the standing class an F1 score of 0.81 (precision 0.91, recall 0.74). The dominant error is standing misclassified as lying (152 frames), occurring mainly during the rapid stand–lie alternation of protocol step 5 and during the bending movements of step 6: in both, the vertical position of the thermal centroid drops well before a horizontal posture is reached, and the centroid-based representation responds to that displacement. The converse error, lying classified as standing, is rare (45 frames, 3.2% of the lying class).

The sit class is retained in the model and in the confusion matrix for completeness only. Sitting was not a scheduled posture in the protocol and is represented by a single labelled frame, so no meaningful metrics can be computed for it. It nevertheless remains an active prediction target and absorbs 27 predictions (16 from lying, 11 from standing) that would otherwise have been assigned to one of the two evaluated classes. Removing the class would require retraining and is left to future work; its presence is noted here so that the 27 frames are not silently unaccounted for.

Where in the room the errors occur. Because a centroid is logged whenever the detector fires, per-class recall can be resolved by distance from the node with no imputation (Table 6). Recall of the standing class is not monotone in distance: it peaks at 0.93 between roughly 2.5 and 3.4 m, falls to 0.70 beyond 3.4 m, and drops to 0.26 between 1.4 and 1.9 m and to 0.10 closer than 1.4 m. Of the near-field failures, 66% and 90%, respectively, are assigned to lying. The lying class shows recall between 0.88 and 1.00 at every distance. Recomputing the bins for tilts of 25 and 35 degrees moves the 1.9 m boundary to 2.2 m and 1.6 m, respectively; as an independent check on the geometry, the model places a standing subject at the far wall at centroid row 71, and the smallest row observed anywhere in the dataset is 70. A comparable asymmetry appears along the horizontal axis (standing recall 0.28 at one edge of the cropped window against 0.91 at the other), but the azimuth of the optical axis within the room was not recorded and it is therefore left uninterpreted.

### 3.3. End-to-End Binary Performance and the Cost of Non-Detection

For the post-fall indicator, the task reduces to a binary decision of whether the person is lying or not. Evaluated end-to-end over all 3034 labelled frames, with non-detections scored as not-lying predictions, the confusion counts are TP = 1333, TN = 1039, FP = 152 and FN = 510. This corresponds to a sensitivity of 0.723 (95% CI 0.637–0.812), specificity of 0.872 (0.828–0.912), precision of 0.898 (0.871–0.924), accuracy of 0.782, balanced accuracy of 0.798, F1 of 0.801 (0.742–0.856) and a Matthews correlation coefficient of 0.582 (0.501–0.665). Restricted instead to the 2010 detected frames, the same quantities are a sensitivity of 0.956, specificity of 0.753, precision of 0.898, F1 of 0.926 and MCC of 0.745 (Table 7).

The gap between the two columns of Table 7 is not a matter of presentation, and its structure needs to be stated plainly. Of the 1039 true negatives, 575 (55.3%) are frames in which the detector simply produced no output while the subject was standing. Of the 510 false negatives, 449 (88.0%) are likewise non-detections rather than misclassifications. The end-to-end specificity of 0.872 and precision of 0.898 are therefore substantially financed by the detector failing to see a standing person, not by the classifier correctly discriminating standing from lying. This inverts the usual trade-off narrative: any improvement in detector recall on the standing class will, all else being equal, reduce the reported specificity and precision, because it converts silent frames into predictions that can be wrong. Conversely, the 0.956 conditional sensitivity of the lying class should not be read as an operationally achieved figure, since it excludes the 449 lying frames the system never saw.

### 3.4. Operating Range of the Confidence Threshold

**Figure 3:**
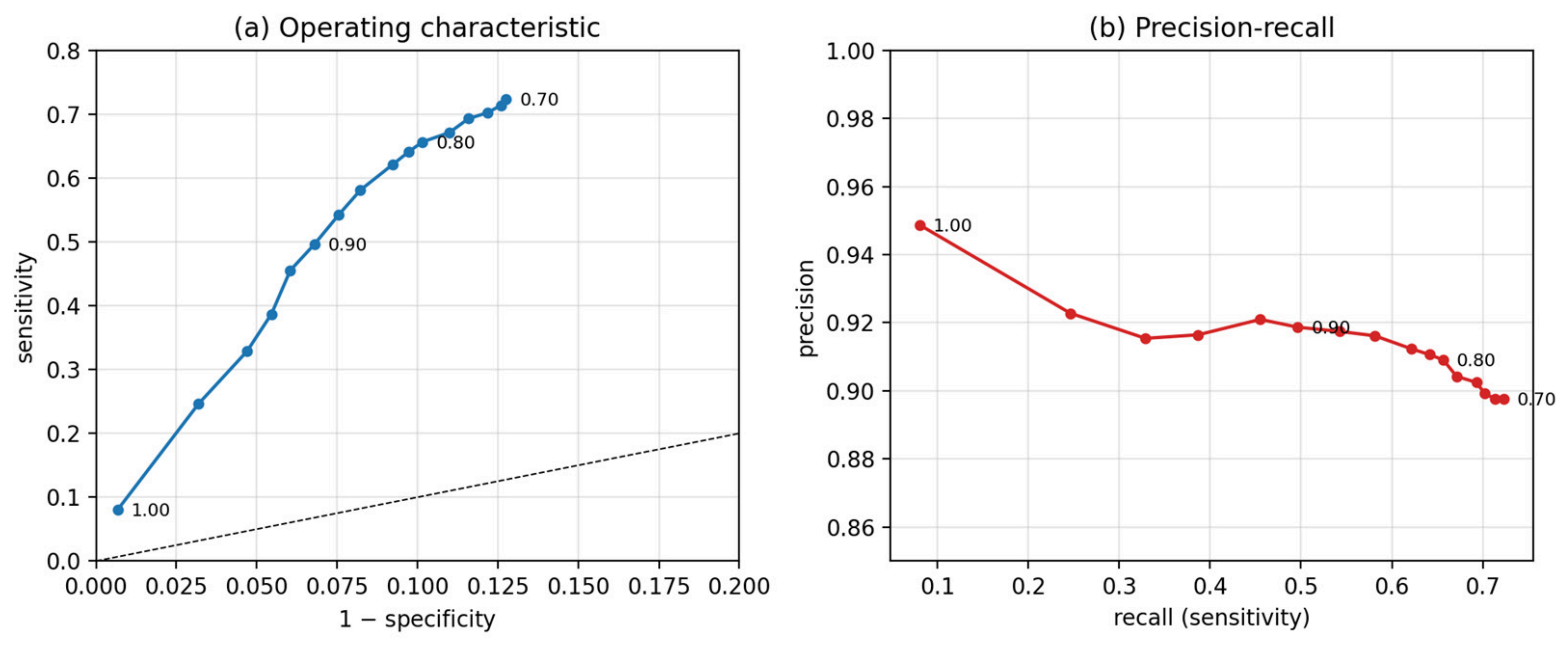
Operating range of the detector confidence threshold over tau in [0.70, 1.00], evaluated end-to-end on all 3034 labelled frames. (**a**) Sensitivity against 1—specificity; (**b**) precision against recall. Labelled points mark tau = 0.70, 0.80, 0.90 and 1.00. The curve is one-sided: the firmware discarded detections below tau = 0.70, so lower thresholds cannot be reconstructed from the recorded data. The dashed diagonal in panel (**a**) marks chance-level performance (sensitivity = 1 − specificity); both axes are truncated to the occupied region.

The deployed detector uses a fixed confidence threshold of MIN_CONF = 0.70 (Section 2.5). Because the firmware discards sub-threshold blobs before logging, thresholds below 0.70 cannot be reconstructed from the recorded data; the sweep presented here therefore spans tau in [0.70, 1.00] and is a one-sided characterization of the operating range. Within that range, the effect is monotone and large (Figure 3, Table 8). Raising tau from 0.70 to 0.98 increases specificity from 0.872 to 0.968 but collapses sensitivity from 0.723 to 0.246, because the additional specificity is purchased entirely by discarding detections: the detection rate on standing frames falls from 51.7% to 5.9% and on lying frames from 75.6% to 24.7%. Both F1 (0.801) and MCC (0.582) are maximized at the deployed threshold of 0.70, and the conditional three-class accuracy is nearly flat across the whole range (0.889 to 0.922), confirming that the threshold governs how often the system speaks rather than how well it classifies when it does. This sweep is the quantitative form of the argument in Section 3.3: within the reachable range, this system cannot be tuned towards higher sensitivity, only towards a quieter and less sensitive operating point. Extending the range downwards would require re-deploying the firmware with a lower MIN_CONF and re-recording, which we identify as the single most informative next experiment.

### 3.5. Event-Level Performance

Applying the episode definition of Section 2.6 to the ground-truth annotations yields 24 lying episodes across the ten subjects. Most subjects contribute two episodes rather than the three suggested by protocol steps 3, 7 and 8, because steps 7 (supine) and 8 (lateral) are performed back-to-back without an intervening standing phase and therefore constitute a single uninterrupted lying bout. Episode durations range from 1.9 s to 48.4 s, and 20 of the 24 episodes last at least 20 s (Table 9).

Against these 24 episodes, the device generated 48 alerts. Under the matching rule of Section 2.6, this gives 12 event true positives, 12 event false negatives and 33 event false positives, i.e., an event-level sensitivity of 0.500 (95% CI 0.273–0.708) and an event-level precision of 0.267 (0.130–0.415), with a median detection latency of 0.24 s across the detected episodes. The false alerts correspond to 117 event false positives per hour of non-lying recorded time; across the whole protocol, the raw alert rate is 48 alerts in 31.4 min, or 92 per hour. These figures are reported in place of the qualitative characterization used previously. An alert rate of roughly one hundred per hour is not moderate for a home-care device: it is the regime in which monitoring systems are muted by their users and subsequently ignored, and it must be treated as a blocking issue for deployment rather than as a tuning detail.

**Figure 4:**
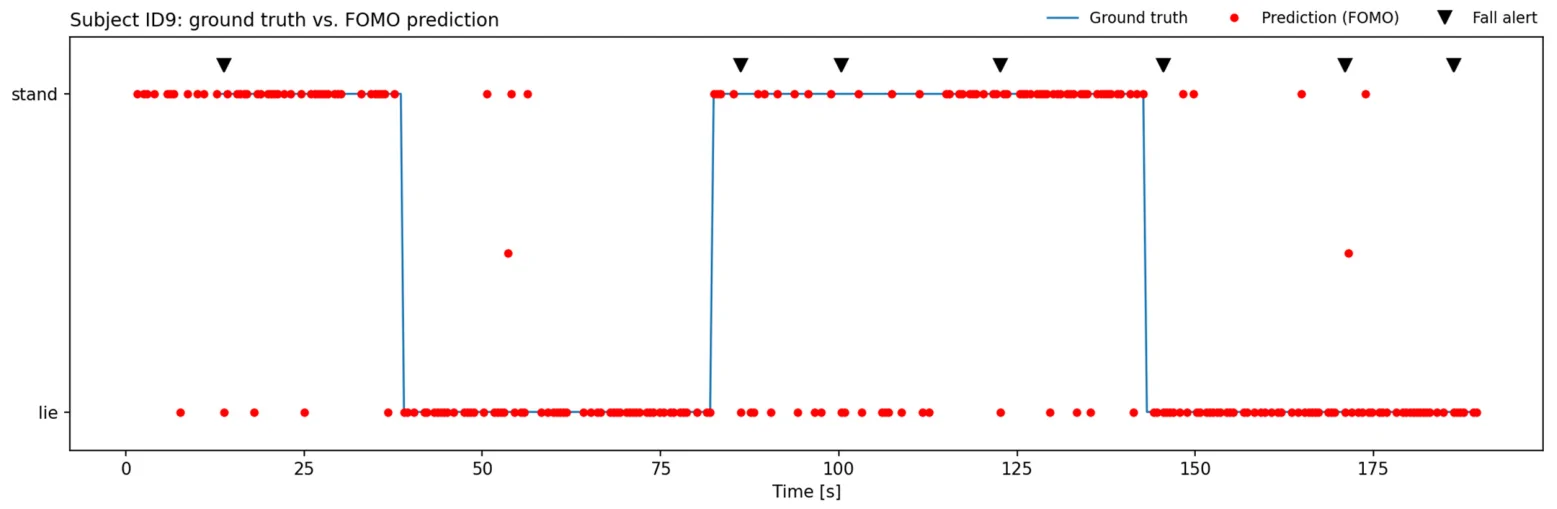
Ground-truth (blue line) and per-frame FOMO predictions (red dots) for subject ID9, selected as the subject whose detection rate (0.669) and conditional accuracy (0.913) are closest to the cohort medians (0.674 and 0.893). Black triangles indicate alert activations. Alerts cluster at stand–lie transitions and during the activities-of-daily-living segment.

Two structural causes account for the false alerts, and both are properties of the alert logic rather than of the classifier. First, as noted in Section 2.5, at 2.1 Hz, the 200 ms confirmation window is satisfied by a single lying frame, so any isolated misclassification during a bend or a transition raises an alert. Second, the 20 alerts occurring during protocol step 5 might be counted as valid responses of the detection logic, since a lying posture is genuinely present at the instant they fire. We do not count them that way. Step 5 is rapid alternation in which the subject stands up again within seconds; in deployment, an alert followed immediately by standing is a false alarm regardless of what the subject was doing at the instant it fired. Under the event definition applied uniformly here, no alert receives credit for coinciding with a transient posture. The interplay of the per-frame predictions, the ground truth and the resulting alert instants is illustrated for a single subject in Figure 4.

Because the alert parameters are firmware constants rather than learned quantities, their effect can be quantified directly on the recorded frames by re-running the state machine of Section 2.5 with different settings (Table 10). Lengthening the confirmation window from the deployed 0.2 s to 2 s, at the unchanged confidence threshold, reduces the reconstructed false-alert rate from 85 to 21 per hour of non-lying time and raises event precision from 0.37 to 0.74, at a median latency cost of 2.9 s. Those four figures all come from the reconstruction and must be read against one another, not against the device figures given earlier in this section; the reconstruction reproduces 40 of the 48 alerts the device emitted, so it under-counts. A latency of a few seconds is immaterial for a long-lie decision, so this remains a favourable trade in the reframed task, but the parameters were selected on the same ten subjects and the resulting figures should be treated as optimistic.

### 3.6. Long-Lie Detection

The event metrics of Section 3.5 treat every lying episode alike, including bouts of under 2 s produced by the alternation phase, and the figures in this section are therefore not comparable to them: they describe a strict subset of the episodes under a different question. For the post-fall indicator that motivates this work, that question is narrower. When a person remains on the floor, does the system flag it, how quickly, and does the alert stay up? Restricting the analysis to the 20 lying bouts of at least 20 s and using the latching alert state of Section 2.5, all 20 bouts were flagged (20/20, 100%). The median latency from bout onset to the first alert was 0.0 s, the 90th percentile was 1.6 s and the maximum was 25.1 s, and the alert was active for a mean of 94.3% of bout duration (Table 11). The 100% figure applies to sustained bouts only and does not describe the system’s behaviour on short episodes.

## 4. Discussion

The goal of this study was to design and experimentally validate a low-cost, privacy-preserving system for detecting the sustained lying posture that follows a fall, based on low-resolution thermal imaging and edge-based artificial intelligence. The results demonstrate that a low-resolution thermal sensor combined with an FOMO-style detector can achieve a three-class frame-level accuracy of 88.9% on the frames in which it produces an output, and an end-to-end binary F1-score of 0.80 over all labelled frames, while operating fully on a microcontroller-class device at approximately 2 Hz. This represents a favourable trade-off for resource-constrained and privacy-sensitive applications, where computational efficiency and deployment simplicity are critical. At the event level, every sustained lying bout of at least 20 s was flagged, with a median latency of 0.0 s and the alert is active for 94.3% of the bout (Table 11), which is the behaviour a long-lie indicator requires. The system achieves this using only a single low-resolution LWIR sensor and without any transmission of visual data, which is particularly relevant for ambient assisted living scenarios.

When compared to previously reported thermal fall-detection systems, the proposed approach occupies a distinct position in the design space (Table 12). Systems based on higher-resolution thermal sensors or non-embedded processing can achieve higher classification accuracy [10,20], but at the cost of increased computational complexity, energy consumption, or the need for data transmission. Multimodal systems that combine thermal imaging with a PIR array or a depth sensor further improve robustness but substantially increase hardware complexity and installation effort [21]. The comparison in the opposite direction must also be stated plainly: most low-cost infrared work uses 8 × 8 to 32 × 24 thermopile arrays, so on both resolution and node cost the present system is the more expensive and the higher-resolution option rather than the cheaper one. What the 19,200-pixel frame buys is enough spatial structure for a single generic centroid detector to separate postures without hand-designed interpolation or blob heuristics, with inference on a Cortex-M-class microcontroller and no host and no network. Cross-study comparison of the headline metrics is indicative only, since cohorts, room geometries and task definitions differ; several of the cited works report the detection of simulated fall events, whereas the present study contains none.

The main residual source of error identified in Section 3 is the limited temporal resolution of the detector. This limitation becomes apparent during rapid stand–lie transitions and bending movements, which produce intermediate body configurations that are difficult to classify reliably. In these situations, the model often assigns the lie label before a fully horizontal posture is reached, reflecting the sensitivity of the centroid-based representation to vertical displacement. We attribute this to the geometry of the representation itself: FOMO predicts class centroids on a coarse output grid, so at a 96 × 96 input the feature that most strongly separates the two classes is the vertical position and elongation of the thermal blob, and both change as soon as the subject begins to descend. Such transient misclassifications need not degrade performance at the event level, but in the configuration evaluated here they do, because the 200 ms confirmation window is short relative to the 470 ms interval at which frames were logged, and the device is observed to raise alerts on isolated lying frames (Section 2.5). Re-running the alert state machine over the recorded frames with a 2 s confirmation window reduces the reconstructed false-alert rate from 85 to 21 per hour of non-lying activity and raises event precision from 0.37 to 0.74, at a median cost of 2.9 s in latency; both ends of that comparison come from the reconstruction of Table 10, which under-counts relative to the alerts the device itself emitted.

Another source of classification ambiguity arises from the geometric configuration of the sensing setup. When a subject moves along the optical axis of the sensor, the projected thermal signature in the 2D image may appear similar for both standing and lying postures. In such cases, the vertical displacement that typically distinguishes these states is not clearly observable, making reliable classification difficult. This limitation is inherent to single-view 2D sensing and is not specific to the proposed model [23]. Our own data localize it (Table 6): the recall of the standing class collapses in the near field of the node while the lying class is unaffected at every distance, and the lost frames are assigned almost entirely to lying. The mechanism is foreshortening: close to a downward-tilted sensor, a standing subject is compressed along the image vertical, the thermal centroid falls towards the floor, and the vertical extent that separates the two postures disappears, whereas a body that is already horizontal loses nothing. The region within roughly 2 m of a corner-mounted node is therefore where a standing person is most likely to be reported as lying, and for a corner-mounted device that is the part of the room a person crosses on entering; this is where the false alerts discussed below originate. In practical deployments, this can be mitigated by appropriate sensor placement, for example, by using an oblique or ceiling-mounted configuration that maximizes observable vertical motion, as in the 2.65 m downward-tilted setup used in this study.

The event-level analysis further illustrates the practical behaviour of the system. Against 24 annotated lying episodes, the device generated 48 alerts, of which only twelve fell inside an episode window; the resulting event-level sensitivity and precision are given in Section 3.5. The false alerts arise during the rapid stand–lie alternation of protocol step 5 and during the bending-to-pick-up segment of step 6; both involve significant vertical displacement of the thermal centroid without a true fall event, and both are challenging cases for posture-based detection. We do not distinguish between them: an alert during a rapid alternation is followed within seconds by the subject standing up again, and in deployment, that is a false alarm regardless of what the subject was doing at the instant it fired. Expressed as a rate, this is of the order of one hundred alerts per hour of non-lying activity. For a home-care device, that is not a moderate rate: it is the regime in which monitoring systems are muted and subsequently ignored, and we regard it as a blocking issue for deployment rather than a tuning detail. It is, however, a property of the alert logic rather than of the classifier, and re-running that logic with a longer confirmation window (Table 10) roughly doubles event precision at a latency cost of about three seconds, which is immaterial for a long-lie decision.

A further observation is the gap between the per-frame recall of the lie class on detected frames and the binary sensitivity evaluated over all labelled frames (Table 5 and Table 7). This difference is largely driven by the detection rate of the model: a third of the labelled frames contained no detection and were therefore treated as negative predictions in the binary evaluation. That rate is not uniform across classes, and the consequence is more serious than a loss of sensitivity. The detector sees a lying person far more often than a standing one, so the majority of the reported true negatives and the great majority of the false negatives are non-detections rather than decisions (Table 4). The reported specificity and precision are therefore substantially financed by the detector failing to see a standing person, and any improvement in its recall on that class would, all else being equal, reduce both. We had expected the detection threshold to provide a design parameter for trading sensitivity against false positives; the sweep in Section 3.4 shows that it does not. Raising the threshold buys specificity only by discarding detections, and both F1 and MCC are maximized at the deployed value. Within the range that can be reconstructed from these recordings, the system can be made quieter, but not more sensitive.

The privacy claim made in this paper rests on those two properties and is deliberately narrow. It is claimed against an adversary with access to the network link and to any data at rest outside the device, but without physical access to the running microcontroller; against that adversary, the properties hold by construction, because the frames never leave the inference loop. We explicitly do not claim that a 160 × 120 LWIR frame is non-identifying. Thermal imagery at this and at lower resolutions has been shown to support person recognition and cross-modality re-identification [24,25], and we have not measured re-identifiability on our own data; a variant of this system that stored or streamed frames would require a separate analysis, which the present design avoids rather than answers, by never producing such a stream. Deployment in the target population raises three further issues that lie outside the technical scope of this study. Consent must be obtained in a form that remains valid under cognitive impairment. The design is consistent with the GDPR principle of data minimization precisely because it retains no imagery, which is an argument available to this architecture and not to a camera-based one. And the monitored person should retain the ability to disable the device, which is a requirement on the installation rather than on the sensor node.

Several limitations of this study should be acknowledged. First, and most importantly, the study contains no fall events: the protocol consists of voluntary, controlled descents, and lying is used throughout as a proxy for a post-fall state, so the reported figures quantify the recognition of that posture and are not comparable with studies that recorded real or staged falls. Public thermal fall datasets exist [26], but none was used here, because they were recorded with different sensors, resolutions and mounting geometries, and because the object of this evaluation is the deployed node end-to-end rather than a model in isolation. Second, the evaluation was conducted on a cohort of ten healthy adult volunteers and did not include older or mobility-impaired individuals; for a thermal posture system, the transfer risk is primarily geometric and thermal rather than dynamic, since kyphosis and a pronounced forward lean reduce exactly the vertical elongation on which the detector depends, and clothing insulation and room temperature alter the apparent thermal signature. Third, all recordings were acquired in a single indoor environment with a fixed camera configuration, and the robustness of the system to variations in ambient conditions—such as temperature, additional heat sources, camera pose, or scene layout—remains to be evaluated. A single corner-mounted node also covers only one room; bathrooms and hallways are high-incidence fall locations that would each require their own node, and whole-dwelling coverage is not addressed here. Fourth, the evaluation set is dominated by lying frames (60.7%), whereas in real monitoring lying is rare; because precision degrades as the positive prior falls, the reported precision is an upper bound on continuous-monitoring performance and the false-alert rate is the more transferable figure. Fifth, the sit class was under-represented in the dataset and its performance metrics are therefore not meaningful, although it remains an active prediction target and absorbs 27 predictions that would otherwise fall to the two evaluated classes. Finally, all annotation was performed by a single annotator, and the dataset size does not allow for statistically robust cross-validation with tight confidence intervals; the reported results should be interpreted accordingly.

Two properties of the acquisition path follow from the design and are recorded here. No visible-spectrum imagery is captured at any point: the sensor is an LWIR microbolometer with no visible-light path. No image data of any kind, whether thermal frame, cropped window or model input, is written to persistent storage or transmitted off the node; the only quantity crossing the device boundary is the alert state.

Future work will focus on improving temporal resolution and robustness while preserving the lightweight nature of the system. The most informative next experiment is to re-deploy with a lower confidence threshold and to log sub-threshold detections, so that the operating curve can be characterized on both sides and the class-dependent miss rate can be addressed directly. Lengthening the alert confirmation window, validated on held-out subjects rather than post hoc, would address the false-alert rate. Recording staged falls onto crash mats is necessary to test the proxy assumption on which the framing rests. Increasing the inference rate towards the native sensor frame rate and incorporating simple temporal filtering (e.g., a moving average of the centroid vertical position) remain promising directions; since the processing period is independent of the number of centroids found and the sensor delivers up to 8.7 Hz, roughly three quarters of the available frame budget is consumed by on-device processing, so this is a software rather than a hardware problem. Extending the dataset to include a broader range of subjects and environments will be essential for further validation. More generally, the presented framework could be extended to detect additional events of interest—such as prolonged immobility or nocturnal bed-exit—enabling a wider range of assistive functions using the same low-resolution thermal sensing platform.

## 5. Conclusions

This study presented a low-cost, privacy-preserving system for detecting the sustained lying posture that follows a fall, based on a 160 × 120 long-wave infrared sensor and an int8-quantized FOMO detector running entirely on a microcontroller-class device, with no imagery stored or transmitted. It was validated on an independent cohort of ten subjects, none of whom contributed training data.

The practical result is that sustained lying is detected reliably: every bout of at least 20 s was flagged, with a median latency of 0.0 s. Two findings qualify this and are, in our view, the more useful contributions. First, the detector produced no output on a third of all labelled frames, and disproportionately on standing frames, so more than half of the reported true negatives and 88% of the false negatives are perception failures rather than decisions; the reported specificity and precision are substantially a product of that miss rate rather than of correct discrimination. Second, the deployed alert logic, whose 200 ms confirmation window is very short relative to the frame interval, produced approximately one hundred false alerts per hour of non-lying activity, and lengthening that window is the single most effective change available without retraining.

No fall, real or simulated, occurs anywhere in this dataset, and no claim is made about the detection of fall events; the cohort is ten healthy adults recorded in a single room with one fixed camera configuration. Within that scope, low-resolution thermal sensing with an on-device centroid detector is a workable, camera-free basis for long-lie detection, and the remaining work lies in the detector’s recall and in the alert logic rather than in the posture classifier.

## Figures and Tables

**Table 1:** Bill of materials of a single sensor node. Prices are list prices at the time of writing and exclude assembly labour and enclosure.

Component	Qty	Unit Cost (USD)
OpenMV Cam RT1062 (NXP i.MX RT1062, Cortex-M7)	1	160
FLIR Lepton 3.1R LWIR module (160 × 120)	1	142
OpenMV Lepton expansion module (breakout)	1	15
Mounting bracket, USB-C power supply, cabling	1	8
Total per node	1	325

**Table 2:** Per-subject dataset statistics and classifier performance. ‘Labelled N’ is the number of frames with an unambiguous static-posture ground truth; ‘Detected N’ is the subset of those in which the FOMO detector produced a centroid above MIN_CONF = 0.70. ‘Detection rate’ is Detected N/Labelled N. ‘3-class acc.’ is the conditional three-class accuracy on the detected subset. ‘Alerts’ are rising edges of the on-device alert state. The Total/mean row reports pooled values; the unweighted mean of the per-subject accuracies is 0.887 and the unweighted mean of the per-subject detection rates is 0.663.

Subject	Duration [s]	Frames (N)	Labelled N	Detected N	Detection Rate	3-Class Acc. (Detected)	Alerts
ID1	178.5	377	281	207	0.737	0.836	7
ID2	191.1	400	309	197	0.638	0.878	3
ID3	190.5	399	308	209	0.679	0.756	4
ID4	188.2	394	303	163	0.538	0.877	8
ID5	188.8	395	303	208	0.686	0.909	4
ID6	191.6	401	310	229	0.739	0.952	4
ID7	189.6	397	306	177	0.578	0.960	3
ID8	187.7	393	302	252	0.834	0.929	4
ID9	189.3	404	308	206	0.669	0.913	7
ID10	187.2	399	304	162	0.533	0.864	4
Total/mean	1882.5	3959	3034	2010	0.662	0.889	48

**Table 3:** Ground-truth distribution over the 3959 recorded frames. Percentages are computed on the 3034 labelled frames; the transition and ignore rows are shown for completeness.

Ground-Truth Class	Labelled Frames	Share [%]
stand	1190	39.2
lie	1843	60.7
sit	1	0.0
Total labelled	3034	100.0
(transition)	673	—
(ignore)	252	—

**Table 4:** Detection rate and outcome decomposition per ground-truth class over the 3034 labelled frames. Each labelled frame falls into exactly one of three outcomes: the detector fired and the class was correct, the detector fired and the class was wrong, or the detector produced no output. Percentages are of the row total.

Ground-Truth Class	Labelled Frames	Detection Rate	Detected and Correct	Detected and Wrong	Not Detected	% Det. and Correct	% Det. and Wrong	% Not Detected
stand	1190	0.517	452	163	575	38.0	13.7	48.3
lie	1843	0.756	1333	61	449	72.3	3.3	24.4
sit	1	1.000	1	0	0	100.0	0.0	0.0
all	3034	0.662	1786	224	1024	58.9	7.4	33.8

**Table 5:** Conditional per-class precision, recall and F1 on the 2010 detected frames. Support is the number of frames of the corresponding ground-truth class within that subset. The sit class is omitted because it is represented by a single labelled frame. These are conditional metrics in the sense of Section 2.6: they exclude the 1024 labelled frames in which the detector produced no output. The corresponding end-to-end figures are given in Section 3.3.

Class	Support	Precision	Recall	F1
stand	615	0.909	0.735	0.813
lie	1394	0.898	0.956	0.926

**Table 6:** Per-class recall resolved by the position of the thermal centroid, over the 2010 detected frames. det_y is measured downwards from the top of the 240 × 240 px window; a larger value means the subject appears lower in the frame and is therefore closer to the corner-mounted sensor. Distances are obtained from the mounting geometry of Section 2.1 under a pinhole model, with the centroid of a standing subject taken at 1.0 m above the floor and that of a lying subject at 0.15 m; they are indicative rather than surveyed, and the sensitivity to the approximate 30-degree tilt is given in the text. No imputation is involved: a centroid is logged whenever the detector fires. The equivalent analysis of the detection rate itself is not possible, because no position is recorded for frames in which the detector produced no output.

Ground-Truth Class	Centroid Row det_y [px]	Distance From Node [m]	Detected Frames	Recall	Fraction Predicted Lie
stand	60–110	>3.4	182	0.703	0.297
stand	110–130	2.5–3.4	239	0.929	0.054
stand	130–150	1.9–2.5	104	0.769	0.221
stand	150–170	1.4–1.9	80	0.263	0.662
stand	170–220	0.8–1.4	10	0.100	0.900
lie	60–110	>5.1	260	0.881	0.881
lie	110–130	3.7–5.1	59	0.881	0.881
lie	130–150	2.8–3.7	492	0.984	0.984
lie	150–170	2.2–2.8	276	0.949	0.949
lie	170–220	1.2–2.2	307	0.997	0.997

**Table 7:** Binary lying-versus-not-lying performance under the two evaluation conventions of Section 2.6. End-to-end scores all 3034 labelled frames, counting a non-detection as a not-lying prediction; conditional scores of only the 2010 frames in which the detector fired. Square brackets give 95% subject-level bootstrap confidence intervals (B = 5000). MCC denotes the Matthews correlation coefficient.

Metric	End-to-End (3034 Labelled Frames)	Conditional (2010 Detected Frames)
True positives (TP)	1333	1333
True negatives (TN)	1039	464
False positives (FP)	152	152
False negatives (FN)	510	61
Sensitivity	0.723 [0.637, 0.812]	0.956
Specificity	0.872 [0.828, 0.912]	0.753
Precision	0.898 [0.871, 0.924]	0.898
Accuracy	0.782	0.894
Balanced accuracy	0.798	0.855
F1	0.801 [0.742, 0.856]	0.926
MCC	0.582 [0.501, 0.665]	0.745

**Table 8:** Effect of the detector confidence threshold tau on end-to-end binary performance over the 3034 labelled frames. Detection rates are reported per ground-truth class. The deployed value is tau = 0.70; values below it cannot be evaluated because sub-threshold detections were discarded by the firmware before logging.

Tau	Detection Rate	Det. Rate (Stand)	Det. Rate (Lie)	Sensitivity	Specificity	Precision	F1	MCC	Cond. 3-Class Acc.
0.70	0.662	0.517	0.756	0.723	0.872	0.898	0.801	0.582	0.889
0.74	0.636	0.488	0.732	0.702	0.878	0.899	0.789	0.568	0.891
0.78	0.595	0.434	0.698	0.671	0.890	0.904	0.770	0.551	0.895
0.82	0.557	0.389	0.665	0.641	0.903	0.911	0.753	0.537	0.901
0.86	0.482	0.298	0.601	0.581	0.918	0.916	0.711	0.501	0.905
0.90	0.402	0.229	0.514	0.496	0.932	0.919	0.645	0.446	0.903
0.94	0.298	0.147	0.396	0.387	0.945	0.916	0.544	0.372	0.905
0.98	0.173	0.059	0.247	0.246	0.968	0.923	0.389	0.284	0.922

**Table 9:** Lying-episode census under the episode definition of Section 2.6 (maximal runs of lying frames, runs separated by less than 2 s merged). Protocol steps 7 and 8 are performed without an intervening standing phase and therefore form a single bout, which is why most subjects contribute two episodes rather than three.

Subject	Recording [s]	Lying Episodes	Total Lying [s]	Shortest [s]	Longest [s]
ID1	178.5	4	85.5	1.9	46.1
ID2	191.1	2	93.6	45.6	48.0
ID3	190.5	2	93.1	45.6	47.5
ID4	188.2	3	71.7	5.7	45.1
ID5	188.8	3	70.6	1.9	45.5
ID6	191.6	2	90.7	42.3	48.4
ID7	189.6	2	92.1	45.6	46.5
ID8	187.7	2	90.2	44.6	45.6
ID9	189.3	2	89.2	42.9	46.2
ID10	187.2	2	86.7	42.7	44.0
Total	1882.5	24	863.4	1.9	48.4

**Table 10:** Event-level performance of the alert state machine of Section 2.5 as a function of its two free parameters, obtained by re-running the state machine over the recorded frames against the 24 annotated lying episodes. These figures are a reconstruction and are not directly comparable with the alerts the device itself emitted (Section 3.5): the reconstruction yields 40 alerts at the deployed settings against the 48 the device logged, because the recorded frame series does not necessarily contain every inference step. Rows should therefore be compared to one another rather than to Section 3.5. The analysis is also post hoc on the evaluation cohort and is not validated on held-out subjects.

MIN_CONF	FALL_CONFIRM [s]	Alerts	Event TP	Event FP	Event FN	Event Sens.	Event Prec.	Event F1	FP/h Non-Lying	Median Latency [s]
0.70	0.2	40	14	24	10	0.583	0.368	0.452	84.8	0.48
0.70	1.0	27	15	11	9	0.625	0.577	0.600	38.9	2.84
0.70	2.0	23	17	6	7	0.708	0.739	0.723	21.2	2.85
0.70	3.0	20	15	5	9	0.625	0.750	0.682	17.7	2.85
0.70	5.0	14	13	1	11	0.542	0.929	0.684	3.5	4.74
0.80	0.2	34	13	20	11	0.542	0.394	0.456	70.7	1.90
0.80	1.0	24	15	8	9	0.625	0.652	0.638	28.3	2.85
0.80	2.0	18	15	3	9	0.625	0.833	0.714	10.6	4.28
0.80	3.0	16	13	3	11	0.542	0.812	0.650	10.6	4.75
0.80	5.0	14	13	1	11	0.542	0.929	0.684	3.5	6.64
0.90	0.2	21	15	6	9	0.625	0.714	0.667	21.2	2.37
0.90	1.0	20	16	4	8	0.667	0.800	0.727	14.1	4.04
0.90	2.0	12	11	1	13	0.458	0.917	0.611	3.5	4.27
0.90	3.0	11	10	1	14	0.417	0.909	0.571	3.5	9.50
0.90	5.0	10	9	1	15	0.375	0.900	0.529	3.5	16.15

**Table 11:** Long-lie performance on the 20 lying bouts of at least 20 s, using the deployed alert configuration. Latency is measured from bout onset to the first alert; alert occupancy is the fraction of the bout during which the latching alert state was active.

Subject	Bouts ≥ 20 s	Mean Bout [s]	Median Latency [s]	Mean Alert Occupancy
ID1	2	39.0	0.23	0.995
ID2	2	46.8	0.48	0.990
ID3	2	46.5	0.48	0.830
ID4	2	33.0	0.00	1.000
ID5	2	34.4	12.53	0.697
ID6	2	45.3	0.00	1.000
ID7	2	46.0	0.00	1.000
ID8	2	45.1	0.00	1.000
ID9	2	44.5	0.92	0.958
ID10	2	43.4	1.87	0.958
All	20	42.4	0.00	0.943

**Table 12:** Positioning against the nearest comparable systems. Node costs are not tabulated because the cited works do not report them; the bill of materials for the present system is given in Table 1. Cross-study comparison of the headline metrics is indicative at best, since the cohorts, room geometries and, most importantly, the task definitions differ: several of these works report detection of simulated fall events, whereas the present study reports recognition of a sustained lying posture and contains no fall events. A dash marks a figure not extracted from the cited work.

Work	Sensor	Pixels	Processing	Subjects	Task/Protocol	Headline Metric
Tateno et al. [10]	IR array 8 × 8	64	off-device (3D-CNN)	16	simulated falls	acc. 98.8%, F1 0.949
Newaz and Hanada [22]	IR array 8 × 8	64	off-device (rule based)	1	activity recognition	not comparable (different task)
Lin and Zhao [20]	IR array 8 × 8	64	off-device	3	occupancy and position	not comparable (different task)
Newaz and Hanada [21]	low-res IR array	64	Raspberry Pi (on node)	2	simulated falls	—
This work	FLIR Lepton 3.1R	19,200 (96 × 96 model input)	Cortex-M7 MCU (on node)	10 (+2 training)	voluntary lying, no falls	cond. acc. 88.9%; end-to-end F1 0.801

## Data Availability

The data presented in this study are available on request from the corresponding author due to as the dataset forms part of a broader ongoing project and is not publicly available.
